# Elevated Exhaled Nitric Oxide in Allergen-Provoked Asthma Is Associated with Airway Epithelial iNOS

**DOI:** 10.1371/journal.pone.0090018

**Published:** 2014-02-28

**Authors:** Abraham B. Roos, Michiko Mori, Reidar Grönneberg, Christina Österlund, Hans-Erik Claesson, Jan Wahlström, Johan Grunewald, Anders Eklund, Jonas S. Erjefält, Jon O. Lundberg, Magnus Nord

**Affiliations:** 1 Department of Medicine, Solna, Respiratory Medicine Unit, Karolinska Institutet, Stockholm, Sweden; 2 Department of Experimental Medical Science, Lund University, Lund, Sweden; 3 Department of Medicine, Division of Hematology, Karolinska Institutet, Stockholm, Sweden; 4 Department of Physiology and Pharmacology, Section of Pharmacological Nitric Oxide Research, Karolinska Institutet, Stockholm, Sweden; 5 Safety Science, Global Regulatory Affairs & Patient Safety, AstraZeneca Global Medicines Development, Mölndal, Sweden; University of Illinois at Chicago, United States of America

## Abstract

**Background:**

Fractional exhaled nitric oxide is elevated in allergen-provoked asthma. The cellular and molecular source of the elevated fractional exhaled nitric oxide is, however, uncertain.

**Objective:**

To investigate whether fractional exhaled nitric oxide is associated with increased airway epithelial inducible nitric oxide synthase (iNOS) in allergen-provoked asthma.

**Methods:**

Fractional exhaled nitric oxide was measured in healthy controls (n = 14) and allergic asthmatics (n = 12), before and after bronchial provocation to birch pollen out of season. Bronchoscopy was performed before and 24 hours after allergen provocation. Bronchial biopsies and brush biopsies were processed for nitric oxide synthase activity staining with nicotinamide adenine dinucleotide phosphate diaphorase (NADPH-d), iNOS immunostaining, or gene expression analysis of *iNOS* by real-time PCR. NADPH-d and iNOS staining were quantified using automated morphometric analysis.

**Results:**

Fractional exhaled nitric oxide and expression of *iNOS* mRNA were significantly higher in un-provoked asthmatics, compared to healthy controls. Allergic asthmatics exhibited a significant elevation of fractional exhaled nitric oxide after allergen provocation, as well as an accumulation of airway eosinophils. Moreover, nitric oxide synthase activity and expression of iNOS was significantly increased in the bronchial epithelium of asthmatics following allergen provocation. Fractional exhaled nitric oxide correlated with eosinophils and iNOS expression.

**Conclusion:**

Higher fractional exhaled nitric oxide concentration among asthmatics is associated with elevated *iNOS* mRNA in the bronchial epithelium. Furthermore, our data demonstrates for the first time increased expression and activity of iNOS in the bronchial epithelium after allergen provocation, and thus provide a mechanistic explanation for elevated fractional exhaled nitric oxide in allergen-provoked asthma.

## Introduction

Allergic asthma is characterized by airway hyperreactivity and variable airflow obstruction caused by an abnormal inflammatory response to foreign antigens, and presence of allergen-specific serum IgE. Alarmingly, the prevalence of allergic asthma is escalating [1]. Enhanced knowledge of the mechanisms contributing to allergic asthma and improved disease monitoring is key to prevent increasing morbidity and mortality, and to limit rising health care costs. Fractional exhaled nitric oxide (FENO) is a validated marker for airway inflammation [2]-[4] that correlates with airway eosinophilia [5], suggesting a particular relevance for asthma management. Recent studies also provide evidence of increased FENO following allergen provocation of allergic asthmatics [6], [7]. However, the mechanisms underlying allergen-induced elevation of FENO remain unknown. The activity of inducible nitric oxide synthase (iNOS/*NOS2*) in the airway epithelium has been suggested to be the most important determining factor for the concentration of FENO in stable asthma [8]–[10]. Thus, we hypothesized that elevated FENO in allergen-induced asthma is associated with increased expression of iNOS.

In this study, we found that elevated FENO in asthmatics after allergen provocation was associated with an increased epithelial staining for nicotinamide adenine dinucleotide phosphate diaphorase (NADPH-d), detecting nitric oxide (NO) synthase activity [11], together with an increased immunoreactivity for iNOS in the bronchial epithelium. Thus, we here for the first time provide a mechanistic explanation for the increased FENO levels in allergen-provoked asthma, underpinning the use of FENO to monitor atopic asthma and improve the care of this common and severe disease.

## Methods

### Study subjects

12 mild atopic asthmatics with allergic-specific IgE to birch pollen (≥2 kU/l; age 22–42 years; 7 females), and 14 non-allergic healthy volunteers (age 23–46, 8 females) were recruited to the Lung and Allergy Clinic at Karolinska University Hospital in Solna, Sweden (Table 1). All healthy volunteers had a negative reaction (≤0.35 kU/l) to a mix of common inhalant allergens (Phadiatop, Immuno CAP System, Phadia AB, Uppsala, Sweden) and all asthmatic subjects were asymptomatic, and used inhaled β2-agonists as needed only. A four week wash-out period for inhaled corticosteroids and a two week wash-out period for anti-histamines, anti-leukotrienes and NSAIDs was applied. None of the patients had experienced an exacerbation or respiratory infection during four weeks prior to sampling. A detailed description of the clinical parameters has been previously published [12]. Participation was voluntary and all subjects provided informed written consent. The protocol and information was approved by the local ethics committee (The Ethical Review Board in Stockholm).

**Table 1:** Clinical characteristics of asthmatics and healthy controls.

	**Asthmatics**	**Healthy controls**
Subjects, *n*	12	14
Sex (F/M)	5/7	8/6
Age (mean, range), years	29 (22–42)	29 (22–46)
FEV1% (mean ± SD)	100±12	105±9
Phadiatop (% positive)	100	All negative
RAST, birch pollen (mean, SD) kU/L	30±29	0
Metacholine challenge PD20 (mean ± SD)	430±558	0
Allergen challenge SQ PD20 (mean ± SD)	861±917	ND
Maximal drop in FEV 1% after allergen challenge (mean ± SD)	31±12	ND

F, female; M, male; FEV1%, forced expiratory volume in one-second – percent of predicted normal; SQ, arbitrary units relating to the concentration of allergen; PD20, provocative dose causing a 20% fall in FEV1; SD, standard deviation; ND, not done; RAST, radioallergosorbent test.

### Lung function tests, measurement of fractional exhaled nitric oxide and fiberoptic bronchoscopy

Expiratory volumes were measured with a dynamic spirometer (Vitalograph®, Buckingham, UK) in a standardised manner. Both forced expiratory volume in one-second (FEV1) and forced vital capacity (FVC) were determined. FENO was measured before, 1 hour after, and 24 hours after bronchial provocation, according to a previously described protocol [13]. Fiberoptic bronchoscopy was performed as described elsewhere [14]. All subjects included in the study underwent a medical examination at least 2 weeks prior to bronchoscopy, including chest-radiographs and blood test. Following a minimum of 3 weeks after the first bronchoscopy, asthmatics were subjected to bronchoprovocation with individually adapted birch pollen extract doses (Aquagen SQ, ALK, Copenhagen, Denmark) as previously described [15], [16]. Provocation was performed outside pollen season. Bronchoprovocation was ended when a drop in FEV_1_≥20% from the post-diluent baseline was observed, as described elsewhere [16]. The second bronchoscopy was performed 24 hours post-allergen provocation. Bronchoalveolar lavage (BAL) was performed as described in detail by others [13].

### Sample preparation

Total and differential cell counts were prepared as previously described [17]. Snap frozen bronchial biopsies collected during bronchoscopy were embedded in OCT (Tissue-Tek, Miles Laboratories, IN) and 10 µm cryo sections were generated. RNA was prepared from cells obtained by bronchial brushing using RNeasy Mini (Qiagen, Hilden, Germany) and RNA-to-cDNA high capacity kit (Applied Biosystems, Carlsbad, CA), was used to transcribe RNA to cDNA. The integrity of RNA was assessed on a 2100 Agilent Bioanalyser (Agilent Technology Inc., Santa Clara, CA) using Agilent RNA 6000 Nano Kit (Agilent). Degraded samples were excluded from the analysis.

### Histological and immunohistochemical staining

Cryo sections where intact airway epithelium could be identified (n = 9) were air dried for 20 minutes at room temperature, and fixed in 1% paraformaldehyde for 10 minutes. Biopsies without identified airway epithelium were excluded from the analysis. NADPH-d staining was performed as described elsewhere [18]. Sections for iNOS immunostaining were rinsed in wash buffer (K8007, Dako, Glostrup, Denmark). Antigen retrieval was performed in a Dako pre-treatment module at 97°C for 20 minutes with antigen retrieval buffer, pH 6.1 (EnVision™ FLEX Target Retrieval Solution, K8005, Dako). Immunostaining for iNOS was performed using EnVision™ Peroxidase/DAB Detection System kit (Rabbit/Mouse K5007, Dako) with an automated slide stainer (Autostainer Plus, DakoCytomation, Glostrup, Denmark) according to instructions provided by the manufacturer. Briefly, endogenous peroxidase activity was blocked with 0.3% hydrogen peroxide for 10 minutes, followed by a blocking step in Dako Protein Block Serum Free solution (X0909) for an additional 10 minutes. Tissue sections were incubated with a primary rabbit polyclonal antibody NO-53 (1∶7000, kind gift from John L. Humes, Merck, USA) directed against a human 6-amino acid C-terminal iNOS peptide for 1 h, followed by incubation with a rabbit polymer/HRP-linked secondary antibody for 30 minutes. 3,3′-diaminobenzidine (DAB) was applied for 10 minutes to detect immunoreactivity. Tissue sections were subsequently counterstained with Mayer's haematoxylin, dehydrated and mounted with Pertex (HistoLab, Gothenburg, Sweden). The specificity of the immunostaining was confirmed by lack of staining after omitting the primary antibody. Staining for NADPH-d or iNOS was performed simultaneously on all sections to avoid variability in staining intensity.

### Histological analysis and quantification

High-resolution digital images of tissue specimens were generated from all sections utilizing a ScanScope Slide Scanner (Aperio Technologies, Vista, CA). Morphometric analyses were performed using Aperio ImageScope v.10.0 software (Aperio Technologies). On each section, intact airway epithelium was manually delineated, and a pixel threshold for positivity was set to only include positive stained pixels (corresponding to iNOS immunoreactivity or NADPH-d staining) using Aperio Positive Pixel Count Algorithm v.9 (Aperio Technologies). For NADPH-d stained slides, the intensity threshold was set to include only strong positive cells (exclude background signal). Samples where intact airway epithelium could not be identified were excluded. Epithelial cells detached from the basal membrane were omitted from the analysis. The percentage of airway epithelium occupied by iNOS or NADPH-d staining was calculated by an investigator blinded to the protocol, using the number of immunopositive pixels above the selected threshold, and the total number of pixels (i.e. immunopositive and negative).

### Real-time PCR analysis

Gene expression analysis of *NOS2* and *NOS3* was performed in duplicates on a 7900HT thermo cycler (Applied Biosystems, Carlsbad, CA). Expression levels were normalized to glyceraldehyde 3-phosphate dehydrogenase (GAPDH). All primers and probes were purchased from Cybergene (Stockholm, Sweden).

### Statistical analysis

Statistical analyses were performed with GraphPad Prism 5 (GraphPad Software, Inc, La Jolla, CA). Mann-Whitney U-test was used for pair-wise comparisons between asthmatics and controls and Wilcoxon matched pairs test when assessing effects of allergen provocation. Spearman's rank correlation test was used for correlation analyses. Differences were considered statistically significant at p<0.05.

## Results

### Higher concentration of FENO in asthmatics

**Figure 1:**
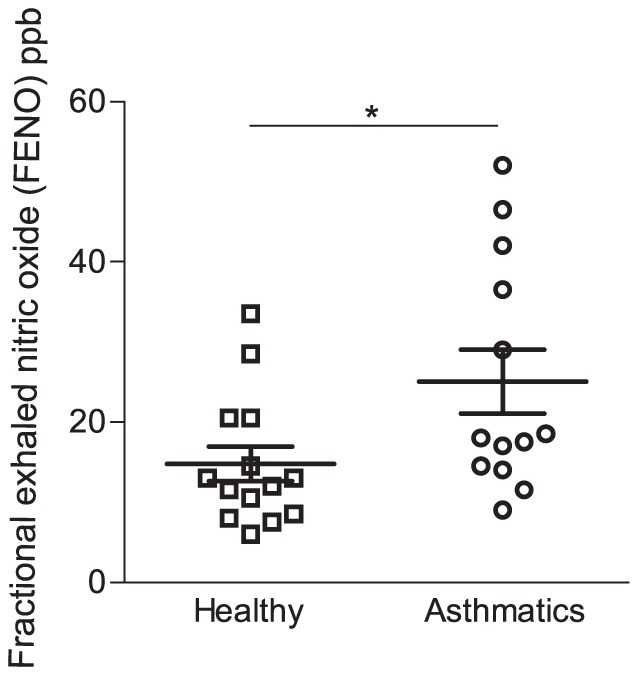
Higher concentration of fractional exhaled nictric oxide (FENO) among allergic asthmatics. FENO was assessed in exhaled breath of healthy controls (n = 14) and asthmatics allergic to birch pollen (n = 12). Bars indicate mean value of all individuals, errorbars indicate SEM. Significant differences between asthmatic individuals and healthy controls were assessed using unpaired t-test. * indicates p<0.05. ppb, parts per billion.

FENO was first measured in healthy controls and compared to the concentration in exhaled breath of un-provoked asthmatics. In agreement with earlier reports (6, 19, 20), significantly higher FENO levels were observed in asthmatics compared to healthy controls (p<0.05, Figure 1). Furthermore, the observed values corresponded well to the previous documentation [19], [20], suggesting that there are only small differences in FENO between mild asthmatics and healthy controls at base-line.

### Higher expression of NOS2 among asthmatic subjects

**Figure 2:**
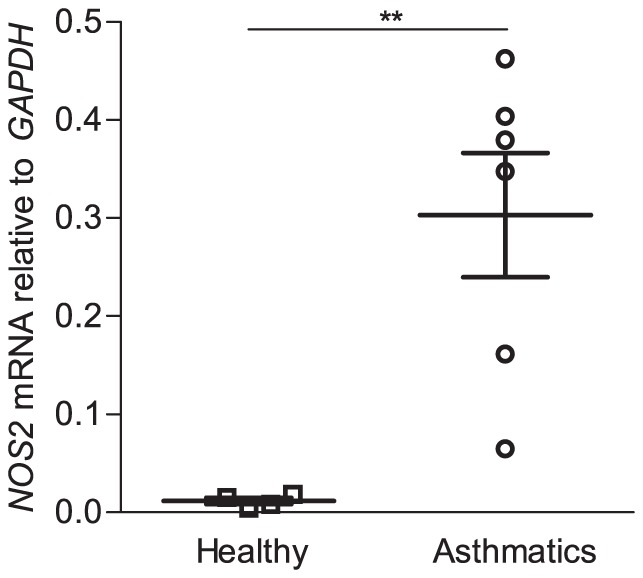
Expression of inducible nitric oxide synthase mRNA (*NOS2*) is increased in the bronchial epithelium of allergic asthmatics. RNA was extracted from cells isolated from bronchial brush biopsies collected during fiberoptic bronchoscopy of healthy controls (n = 4), and allergic asthmatics (n = 6). The integrity of RNA was assessed, and degraded samples were excluded from the analysis. Gene expression of *NOS2* (coding for iNOS) relative to glyceraldehyde 3-phosphate dehydrogenase (*GAPDH*) was analyzed by semi-quantitative real-time PCR. Bars indicate means, errorbars indicate SEM. Significant differences were assessed using unpaired t-test. ** indicates p<0.01.

We next assessed the mRNA expression of *NOS2* (coding for iNOS) and *NOS3* (eNOS) in RNA extracted from bronchial brush biopsies, which consist of >90% epithelial cells [21]. No differences in *NOS3* expression were observed (data not shown). In contrast, the epithelial mRNA expression of *NOS2* was significantly higher in asthmatics compared to healthy controls (p<0.01, Figure 2). A correlation between the expression of *NOS2* and the concentration of FENO was, however, not detected (data not shown).

### FENO is elevated in asthmatic subjects following bronchial allergen provocation

**Figure 3:**
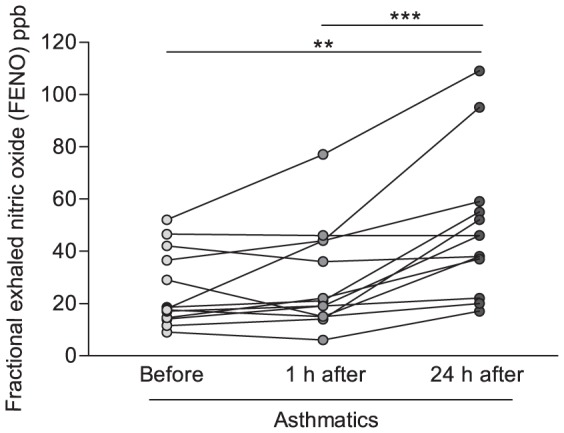
Elevated fractional exhaled nitric oxide (FENO) in allergic asthmatics following bronchial allergen provocation. FENO was assessed in exhaled breath of asthmatics allergic to birch pollen (n = 13), before (light grey), 1 hour after (grey) and 24 hours after (dark grey) bronchial provocation with birch pollen. Bars indicate mean value of all individuals. Significant differences were assessed using paired t-test. ** indicates p<0.01. ppb, parts per billion.

**Figure 4:**
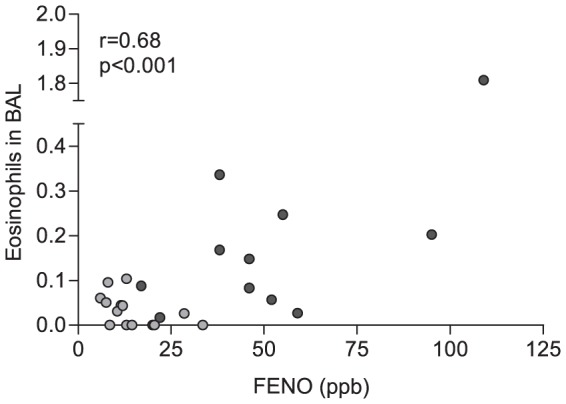
Increased airway eosinophils correlates with elevated fractional exhaled nitric oxide (FENO). Bronchoalveolar lavage (BAL) fluid was collected during fiberoptic bronchoscopy in allergic asthmatics (n = 13), before (light grey) and after allergen provocation (dark grey). BAL eosinophils were enumerated and correlated to FENO using Pearson correlation test of linear regression. ppb, parts per billion.

Allergic asthmatics was subsequently bronchial-provoked to birch pollen, and the concentration of FENO was evaluated. Three asthmatics developed a late phase reaction [12], although the response in these individuals did not appear atypical. The concentration of FENO was significantly elevated 24 hours after allergen challenge (p<0.01, Figure 3), in support of previous findings [6], [7]. As nitric oxide is known to promote bronchodilation [22], elevated FENO after bronchial provocation may be associated with improved lung function. Lung function was, however, significantly depressed 24 hours after birch pollen provocation (Figure S1), and no correlation between the concentration of FENO and FEV1 was observed (data not shown). To evaluate whether airway eosinophils associates with FENO in allergen-provoked asthma, as has been suggested by others [23] we next performed correlation analysis. Airway eosinophils were significantly increased by allergen provocation (data reported by Torregrosa Paredes et al.) [12]. Moreover, in support of previous documentation, a positive correlation between BAL eosinophils and FENO was observed (r = 0.68, p<0.001, Figure 4).

### Increased epithelial NO synthase activity in allergen-provoked asthmatics

**Figure 5:**
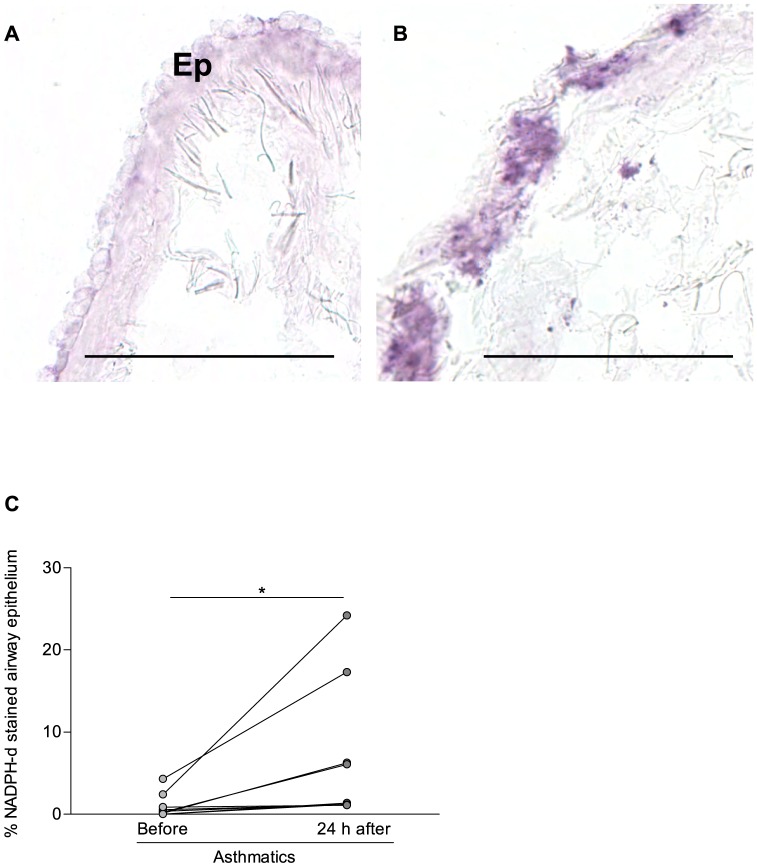
Activity of nitric oxide (NO) synthases is increased in the bronchial epithelium of allergic asthmatics following allergen provocation. Bronchial biopsies were collected during fiberoptic bronchoscopy of allergic asthmatics (n = 9), before (light grey) and 24 hours after allergen provocation (dark grey). Representative image of the epithelial (Ep) activity of NO synthases was assessed by nicotinamide adenine dinucleotide phosphate-diaphorase (NADPH-d) staining (purple) in bronchial biopsies from allergic asthmatics A) before and B) after allergen provocation. C) Morphometric analysis was performed. Samples where intact bronchial epithelium could not be identified were omitted from the analysis. Epithelial cells detached from the basal membrane were excluded. Percentage of bronchial epithelium stained positive for NAPDH-d. Significant differences were assessed using paired t-test. Scale bars indicate 200 µm. * indicates p<0.05.

The molecular source of elevated FENO in allergen-provoked asthma has not been determined, although NO synthases in the airway epithelium have been suggested to influence FENO in stable asthma [8]–[10]. To investigate the origin of the increased FENO in allergen-provoked asthmatics, we next assessed NO synthase activity and iNOS protein expression in the bronchial epithelium. The activity of NO synthases was first addressed by performing NADPH-d staining [11], [18] on bronchial biopsies. The most pronounced staining was detected in the airway epithelium of asthmatics following allergen provocation, with diffuse cytoplasmic localization (Figure 5A–B and Figure S2). We next quantified the NADPH-d staining in the bronchial epithelium. While no significant difference in staining was observed between healthy controls and asthmatic subjects at baseline (data not shown), the percentage of NADPH-d positive epithelium was increased in the airways of asthmatics following allergen provocation (p<0.05), suggesting increased activity of NO synthases in the bronchial epithelium post-provocation. In addition, we observed a weak correlation between the percentage of NADPH-d positive airway epithelium and FENO, although the model only approached significance (r = 0.45, p<0.061, Figure S3). Taken together, this supports epithelial NO synthase as a possible source of increased FENO levels following allergen provocation.

### Elevated FENO after bronchial allergen provocation in asthmatics correlates with increased expression of airway epithelial iNOS

**Figure 6:**
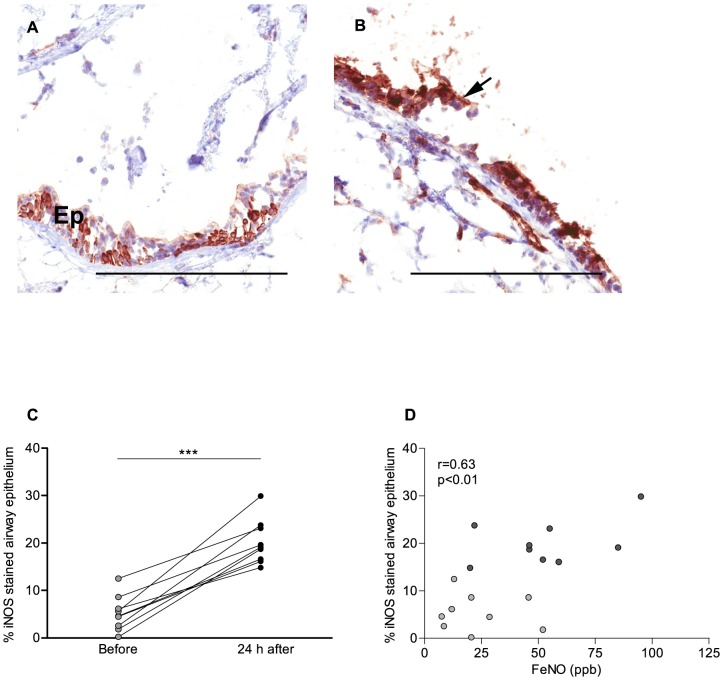
Expression of inducible nitric oxide synthase (iNOS) is increased in the bronchial epithelium of allergic asthmatics following allergen provocation. Bronchial biopsies were collected during fiberoptic bronchoscopy of allergic asthmatics (n = 9), before (light grey) and 24 hours after allergen provocation (dark grey). Representative image of the epithelial (Ep) expression of iNOS (brown color) was assessed by immunohistochemistry in bronchial biopsies from allergic asthmatics A) before and B) after allergen provocation. Cell nuclei were counterstained with Mayer's haematoxylin (blue stain). Morphometric analysis was performed. Samples where intact bronchial epithelium could not be identified were omitted from the analysis. Epithelial cells detached from the basal membrane were excluded (indicated by arrow). C) Percentage of bronchial epithelium positive for iNOS immunoreactivity and D) correlation analysis of the percentage of positive epithelium and fractional exhaled nitric oxide (FENO) using Pearson correlation test of linear regression. Significant differences were detected using paired t-test. Scale bars indicate 200 µm. *** indicates p<0.001.

The NADPH-d staining is not specific for any individual NOS isoform (eg. iNOS, endothelial (e)NOS and neuronal (n)NOS) [18]. Therefore, and to address whether iNOS in the airway epithelium is elevated following bronchial-allergen provocation of asthmatics, the protein expression of airway epithelial iNOS was next assessed by immunohistochemical detection in bronchial biopsies (Figure 6). In line with a previous observation [24], apical and cytoplasmic iNOS immunostaining was readily detected in the bronchial epithelium of healthy controls (Figure S4), as well as in asthmatic subjects (Figure 6A–B). In parallel with the NO synthase activity, the percentage of airway epithelium staining positive for iNOS was similar in healthy controls and un-provoked asthmatics (data not shown). In allergen-provoked asthma, however, a significantly higher proportion of the airway epithelium stained positive for iNOS compared to stable asthma (Figure 6 C). A significant increase in *NOS2* mRNA was, however, not detected in asthmatics following provocation (data not shown). However, in further support of a role for airway iNOS in the FENO elevation following allergen provocation, a positive correlation was observed between percentage of positive iNOS-stained epithelium and FENO (Figure 6D, r = 0.63, p<0.01).

## Discussion

In this study, we hypothesized that the elevated FENO in allergen-provoked asthma is associated with increased NO synthase activity and expression of iNOS. A large body of evidence support FENO as a useful marker for airway inflammation [2]–[4]. Furthermore, FENO is associated with asthma exacerbation among children [25], providing further support of FENO as a valuable non-invasive tool to monitor disease that can be used to limit asthma-related morbidity and mortality, as suggested elsewhere [5]. Although previous studies have reported increased FENO in allergen-provoked asthmatics [6], [7], [26] and enhanced our understanding of allergen-induced asthma, the mechanisms behind the elevated FENO has not been fully addressed. Lower pH in the airways during asthma exacerbation has been proposed as a mechanism contributing to increased FENO by non-enzymatic reduction of nitrite [27]. This has been challenged by Rolla and colleagues, who found that allergen-induced FENO is not explained by airway acidification, and suggested increased activity of NO synthases as a probable mechanism [7].

Within this study, elevated FENO was observed together with increased NO synthase activity and iNOS expression in the bronchial epithelium following allergen provocation of allergic asthmatics. Based on the current results, it is reasonable to conclude that an elevated expression of iNOS in the bronchial epithelium is responsible for the observed increase in NO synthase activity, as NADPH-d staining is a specific histochemical marker for cells producing NO [11]. Intervention studies using pharmacological inhibitors of iNOS has demonstrated reduced FENO levels in healthy controls [28], as well as asthmatics, before and after allergen provocation [26], indicating an important role for iNOS in generation of NO. The study by Singh et al. documented the staining intensity of iNOS in the airway mucosa post-allergen challenge, although no biopsies were collected at base-line [26]. The study also reported that there is no decrease in airway hyperreactivity or inflammation with the specific iNOS inhibitor, challenging the benefit of inhibiting NO production. This may, however, be related to the comparable induction of FENO by allergen provocation with iNOS inhibition or placebo treatment [26], suggesting that base-line inhibition of iNOS and FENO, which was targeted in the intervention study is of less relevance.

Contrasting to the increase in iNOS following allergen provocation, the stable *NOS2* mRNA levels reported here may be related to the relatively late time point (24 hours post-provocation) of the second bronchoscopy, where increased activity and expression of iNOS were detected, while mRNA expression may have returned to base-line levels. A more detailed time-course is required to determine whether this is the case. As a majority of RNA samples were degraded, and the sample size was significantly decreased, the power of the analysis is limited.

Higher FENO and iNOS mRNA expression was observed in un-provoked asthmatics compared to healthy controls within this study. Higher iNOS expression or NOS activity was, however, not detected, possibly as a consequence of insufficient sensitivity of the methology used. Alternatively, the elevated FENO in asthmatics under basal conditions may predominantly arise from a source proximally or distally to the site where the biopsy was taken in this study. Recent evidence suggests that NO levels are higher in the distal airways compared to the more proximal parts [29], although others have documented an important role for the entire conducting airways in NO generation [30]. Our current data support that the proximal airway epithelium contributes to the elevated FENO in allergen-induced asthma, as the biopsies analyzed in this study were collected from the proximal cristae. The relative contribution of the distal airways to the elevated FENO in allergen-provoked asthma, however, remains to be addressed. Other investigators have reported elevated FENO, as well as higher iNOS expression in severe (non-allergic) asthma compared to healthy controls. In contrast to our current findings, elevated FENO was not detected in mild or moderate asthma [31], suggesting that patients with allergic asthma display higher FENO compared to non-allergic asthmatics, who may exhibit FENO comparable to healthy controls. Further studies with all patient groups are, however, needed to confirm this. Differences in age, atopy, gender and pharmacological therapy between healthy controls and mild-moderate asthmatics in the previous study by Yamamoto et al. [31], may also influence the outcome, as all these factors are known to affect FENO and/or iNOS expression [32].

In our current study, the healthy volunteers were not allergen-provoked. While we acknowledge that this represents a limitation, an earlier study reported that allergens fail to elevate FENO in both allergic as well as non-allergic subjects without airway symptoms [33].

To conclude, in the current study we assessed FENO and investigated the activity of NO synthases and expression of iNOS in stable and allergen-provoked asthma. We found an association of the elevated FENO levels after allergen provocation with both NO synthase activity and protein expression of iNOS in the bronchial epithelium, providing for the first time a potential mechanistic explanation for the increased levels of FENO after allergen-provocation of asthmatics. Thus, our results implicate iNOS signaling in allergen-induced asthma exacerbation, and suggest that FENO is an important tool to monitor allergic asthma that may be used to limit asthma-related morbidity and mortality.

## Supporting Information

Figure S1 Depressed lung function in allergic asthmatics following allergen provocation. Forced expiratory volume in one-second – percent of predicted normal (FEV1%) was measured in asthmatics allergic to birch pollen (n = 12) before and 24 hours after birch pollen provocation. Bars indicate mean value of all individuals, errorbars indicate SEM. Significant differences between asthmatic individuals and healthy controls were assessed using unpaired t-test. ** indicates p<0.01.
(EPS)

Figure S2 Epithelial (Ep) activity of NO synthases of bronchial mucosa from a healthy control subject. Nicotinamide adenine dinucleotide phosphate-diaphorase (NADPH-d) staining (purple) was performed in bronchial biopsies obtained during fiberoptic bronchoscopy. Scale bar indicate 200 µm.
(EPS)

Figure S3 Correlation of the percentage of nicotinamide adenine dinucleotide phosphate-diaphorase (NADPH-d) positive epithelium and fractional exhaled nitric oxide (FeNO). Bronchial biopsies from allergic asthmatics (n = 9), before (light grey) and 24 hours after allergen provocation (dark grey) collected during fiberoptic bronchoscopy was stained for NAPDH-d. FeNO was measured before and after allergen provocation. Correlation analysis was performed using Pearson correlation test of linear regression.
(EPS)

Figure S4 Epithelial (Ep) expression of inducible nitric oxide synthase (iNOS) in bronchial mucosa from a healthy control subject. iNOS immunodetection (brown) was performed on bronchial biopsies obtained during fiberoptic bronchoscopy. Slides were counterstained with hematoxylin (blue). Scale bar indicate 200 µm.
(EPS)
